# Impact of an alternative steroid on the relative bioavailability and bioequivalence of a novel versus the originator formulation of abiraterone acetate

**DOI:** 10.1007/s00280-017-3360-3

**Published:** 2017-07-10

**Authors:** Azra Hussaini, Anthony J. Olszanski, Cy A. Stein, Bill Bosch, Paul Nemeth

**Affiliations:** 1 PAREXEL Early Phase Clinical Unit, Harbor Hospital, 3001 South Hanover Street, 7th floor, Baltimore, MD 21225 USA; 2 Medical Oncology, Fox Chase Cancer Center, 333 Cottman Avenue, Philadelphia, PA 19111 USA; 3 City of Hope, 1500, East Duarte Road, Duarte, CA 91010 USA; 4 Churchill Pharmaceuticals LLC, 3602 Horizon Drive, Suite 160, King of Prussia, PA 19406 USA

**Keywords:** Abiraterone acetate, Bioavailability, Bioequivalence, Methylprednisolone, Prednisone, SoluMatrix Fine Particle Technology™

## Abstract

**Purpose:**

The originator abiraterone acetate (OAA) formulation is used for the treatment of metastatic castration-resistant prostate cancer (mCRPC). This study evaluated the bioavailability and bioequivalence of a novel formulation, abiraterone acetate fine particle (AAFP), versus OAA on a steady-state background of steroids.

**Methods:**

Thirty-seven healthy male subjects were randomized in a crossover design to receive methylprednisolone (4 mg twice daily) or prednisone (5 mg twice daily) for 12 days in Period 1. On Day 11 of Period 1, subjects given methylprednisolone received a single dose of AAFP 500 mg, and subjects given prednisone received a single dose of OAA 1000 mg under fasted conditions. After a 2-week steroid washout period, subjects received the alternate treatments in Period 2.

**Results:**

There were no statistical differences regarding area under the curve (AUC) and maximum concentration (*C*_max_) between AAFP and OAA. The bioavailability of abiraterone from AAFP versus OAA by geometric mean ratio was AUC_0–∞_, 95.9% (90% confidence interval [CI] 86.0–106.9); AUC_0–*t*_, 99.2% (88.7–110.9); and *C*_max_, 116.8% (102.2–133.4). The coefficient of variation (CV) was smaller for AAFP versus OAA (AUC_0–∞_, CV 44.23 vs. 55.61%; AUC_0–*t*_, 45.17 vs. 58.16%; *C*_max_, 54.55 vs. 65.65%, respectively). Both treatments were safe and well tolerated.

**Conclusions:**

AAFP plus methylprednisolone provided abiraterone exposure that was comparable to OAA plus prednisone with respect to *C*_max_ and AUC. Less drug exposure variability was observed with AAFP compared with OAA. Reduced pharmacokinetic variability may positively influence clinical outcomes and warrants further study in mCRPC patients.

## Introduction

Prostate cancer growth is stimulated by androgens. This has led to the development of therapies that inhibit androgen synthesis or action. Abiraterone is an irreversible inhibitor of 17α-hydroxylase/C17, 20-lyase (CYP17A1) [1], a key enzyme in androgen synthesis, and it is approved for use in patients with metastatic castration-resistant prostate cancer (mCRPC). In clinical trials, in patients with mCRPC, abiraterone acetate (AA) reduced circulating testosterone levels to <1 ng/dL [2] in many patients and improved survival rates [3, 4].

One of the side effects of CYP17A1 inhibition is a decrease in cortisol levels and a compensatory increase in adrenocorticotropic hormone (ACTH). Excess ACTH, in turn, leads to an accumulation of steroids with mineralocorticoid properties upstream of CYP17A1 (reviewed by Auchus et al.) [5]. Glucocorticoids (e.g., prednisone) act by suppressing ACTH secretion from the anterior pituitary gland [5]. Prednisone has been used in conjunction with OAA to obviate the mineralocorticoid excess associated with CYP17A1 inhibition, and prevent the resultant side effects, such as hypokalemia, hypertension, and fluid retention [5–8]. Prednisone is the steroid specified in the FDA approval for concomitant administration with OAA [9]. It is also a weak inducer of cytochrome P450 3A4 (CYP3A4) [10], whereas abiraterone is a substrate of CYP3A4 [9].

Methylprednisolone is another frequently used steroid in cancer therapies. A dose of 8 mg/day has been used in combination with docetaxel in men with mCRPC [11]. It is a CYP3A4 substrate [12] that also differs structurally from prednisone. A 5-mg dose of prednisone and a 4-mg dose of methylprednisolone have equivalent glucocorticoid effects, although methylprednisolone has lower mineralocorticoid activity compared with prednisone [13].

Clinical pharmacokinetic evaluations of OAA have shown that abiraterone drug exposure approaches saturation and does not significantly increase beyond the OAA 1000-mg dose [9]. Additionally, a large variability in abiraterone exposure was reported in healthy subjects and in mCRPC patients [8, 10]. In patients, the mean area under the curve (AUC) of OAA at steady state (±standard deviation) is 1173 ± 690 ng·h/mL, with an intersubject variability of 64% [10]. In addition, OAA has a significant food effect; administering OAA shortly after a high-fat meal markedly increases abiraterone absorption compared with administration in the fasted state [10].

New drug manufacturing processes can overcome some of the limitations in drug bioavailability and exposure variability. AA fine particle (AAFP) is a novel proprietary formulation of AA utilizing SoluMatrix Fine Particle Technology™, which is licensed from iCeutica, Inc., to Churchill Pharmaceuticals LLC, for use with AAFP. This formulation was designed to improve the oral bioavailability of abiraterone compared with the OAA formulation and to reduce food effects. In a previous companion study of healthy male subjects, AAFP 500 mg was shown to be bioequivalent to OAA 1000 mg using the 80–125% limit rule for *C*_max_ and AUC for bioequivalence, when taken under fasted conditions and in the absence of steroids [14]. In addition, in another previous study, AAFP was found to have approximately 50% less food effect [15] than what has been reported for OAA [10].

The present study was conducted to evaluate the effect of using an alternative steroid to prednisone (methylprednisolone) on the bioequivalence and variability of abiraterone drug levels following administration under fasted conditions of AAFP in comparison with OAA plus prednisone.

## Materials and methods

This Phase I clinical study was conducted between March 23, 2015, and June 18, 2015, at a single study center in the United States (PAREXEL International, Early Phase Clinical Unit, 3001 South Hanover Street, Baltimore, Maryland 21225, USA). The study complied with the International Conference on Harmonisation, Good Clinical Practice guidelines, and the Declaration of Helsinki. All subjects provided written informed consent before any treatment was initiated. The protocol was reviewed and approved by an institutional review board (Aspire IRB, 11491 Woodside Avenue, Santee, California, 92071, USA).

### Study population

Healthy male subjects aged 18–50 years with a body mass index between 18 and 30 kg/m^2^ and a body weight of at least 50 kg were eligible. Subjects included in the study were required to be in good health based on the results of a physical examination, vital signs, electrocardiography, and clinical laboratory testing (hematology, biochemistry, and urinalysis). Subjects were excluded from the study if they had a history of diabetes or immunosuppression, were susceptible to the psychological effects of steroids, used prescription medication, or received a positive test result for drugs of abuse or alcohol at the screening visit.

### Study design

This was a 2-period, randomized, crossover, open-label, 2-treatment, Phase I study in 37 healthy male volunteers. In Period 1, subjects were admitted at the study center the evening before the first scheduled steroid dose. The following morning (Day 1), subjects were randomly assigned to 1 of 2 treatment regimens: Treatment A consisted of 12 days of methylprednisolone [4 mg twice daily (BID)] with a single dose of AAFP 500 mg on Day 11; Treatment B consisted of 12 days of prednisone (5 mg BID) with a single dose of OAA 1000 mg on Day 11. Twelve days of steroid administration were chosen to achieve a pharmacodynamic steady state and to allow for any enzyme induction that might occur. Subjects began twice-daily dosing with the assigned steroid on Day 1. On the evening of Day 10, all subjects underwent a minimum 10-h overnight fast. The following morning (Day 11), subjects received a single dose of steroid and a single dose of either AAFP 500 mg or OAA 1000 mg. The second dose of steroid was administered later that evening. Subjects continued to receive their assigned steroid twice daily on Day 12, and following the 48-h collection of pharmacokinetic samples, were discharged from the study site the following morning (Day 13).

In Period 2, subjects returned to the center following a 14-day washout period (from dose to dose). The same study procedures were performed as in Period 1; however, the subjects followed the order of their randomly assigned treatment sequences and “crossed over” to the other treatment. Blood samples for determination of AA plasma concentrations were collected predose (−0.75) and at 0.25, 0.5, 1, 1.5, 2, 3, 4, 6, 8, 12, 24, 36, and 48 h after dosing of AAFP or OAA in each study period.

AAFP tablets were manufactured by Mayne Pharma Group, Ltd, for Churchill Pharmaceuticals LLC. OAA tablets, prednisone, and methylprednisolone were obtained commercially.

### Pharmacokinetic analysis

The pharmacokinetic population included subjects who were dosed with both AAFP on a background of methylprednisolone (4 mg BID) and OAA on a background of prednisone (5 mg BID) under fasted conditions. The following plasma pharmacokinetic parameters were determined: the AUC from time 0 to the time (*t*) of the last quantifiable concentration (*C*_*t*_) (AUC_0–*t*_), calculated by the linear trapezoidal method; the apparent elimination rate constant (*K*_e_), determined by linear regression of the terminal points of the log-linear concentration–time curve; the AUC from time 0 to infinity (AUC_0–∞_), approximated by linear trapezoidal summation and extrapolated to infinity by the addition of *C*_*t*_/K_e_; the maximum measured plasma concentration (*C*_max_); the time to maximum measured plasma concentration (*T*_max_); and the apparent terminal elimination half-life (*T*_½_), calculated as log_e_(2)/*K*_e_ or 0.693/*K*_e_.

### Safety assessments

All subjects who received at least 1 dose of AAFP or OAA were included in the safety population. Assessments included physical examinations, vital signs, electrocardiography, clinical laboratory testing (hematology, biochemistry, and urinalysis), and adverse event (AE) assessments. AE assessments and concomitant medications were assessed throughout the clinical study.

### Sample size

The sample size estimation of 30 subjects for this study was based on the power approach of comparative analysis, since the study’s primary analysis involved evaluating the differences in pharmacokinetic parameters between dosing conditions. Accounting for a dropout rate of 20% over the study duration and a balanced 2-treatment crossover design, the investigators planned to randomize and dose 38 subjects without replacement.

### Statistical analyses

For the primary endpoint, analysis of variance (ANOVA) for a 2-treatment crossover design was employed to examine the differences in the rate and extent, as indexed by *C*_max_ and AUC, of drug absorption between the 2 treatment regimens for single doses of AAFP 500 mg (test) and OAA 1000 mg (reference). The ANOVA model included sequence, subject-within-sequence, period, and regimen. The sequence effect was tested using the subject-within-sequence effect, and all other effects were tested using the residual error of the model. A null hypothesis of zero difference in a parameter between each test and reference condition was assessed at the 0.05 significance level, with the alternative hypothesis of nonzero differences. The pharmacokinetic parameters of *T*_max_, *T*_½_, and *K*_e_ were compared for test versus reference regimens using the nonparametric Wilcoxon signed rank test.

To assess the secondary endpoints, bioequivalence, AUC, and *C*_max_ parameters were analyzed on a log scale, using the same model outlined for the primary analysis, to assess bioequivalence of AAFP 500 mg versus OAA 1000 mg. The 2 one-sided *t* test hypotheses were tested at the 0.05 significance level by constructing 90% confidence intervals (CI) for the geometric mean ratios. Bioequivalence was concluded if the 90% CIs of the ratio were within 0.80–1.25 for AUC and *C*_max_ parameters.

## Results

### Patient disposition

Thirty-seven subjects received the treatment in Period 1. One patient who received AAFP in Period 1 was lost to follow-up, leaving 36 patients who completed both treatment periods. Subject baseline characteristics and demographics are shown in Table 1.

**Table 1:** Subject baseline characteristics and demographics

	Treatment sequence		All subjects treated *N* = 37	All subjects completed *N* = 36
	AB *n* = 19	BA *n* = 18		
Baseline characteristics				
Age (years)				
Mean	38.1	34.4	36.3	36.4
SD	8.2	9.1	8.7	8.8
Height (cm)				
Mean	177.6	176.7	177.1	177.2
SD	5.5	7.1	6.3	6.3
Weight (kg)				
Mean	83.6	80.3	82.0	82.0
SD	10.7	10.5	10.6	10.8
BMI				
Mean	26.5	25.7	26.1	26.1
SD	2.5	3.0	2.8	2.8
Demographics				
Ethnicity				
Hispanic/Latino	3 (15.8%)	2 (11.1%)	5 (13.5%)	5 (13.9%)
Not Hispanic/Latino	16 (84.2%)	16 (88.9%)	32 (86.5%)	31 (86.1%)
Race				
White	10 (52.6%)	6 (33.3%)	16 (43.2%)	15 (41.7%)
Asian	1 (5.3%)	0	1 (2.7%)	1 (2.8%)
Black/African American	8 (42.1%)	12 (66.7%)	20 (54.1%)	20 (55.6%)

*AAFP* abiraterone acetate fine particle, *BID* twice daily, *BMI* body mass index, *N* number of subjects included, *OAA* originator abiraterone acetate, *SD* standard deviation

Treatment A: AAFP 500 mg + methylprednisolone (4 mg BID) under fasted conditions

Treatment B: OAA 1000 mg + prednisone (5 mg BID) under fasted conditions

### Pharmacokinetic parameters

**Fig. 1:**
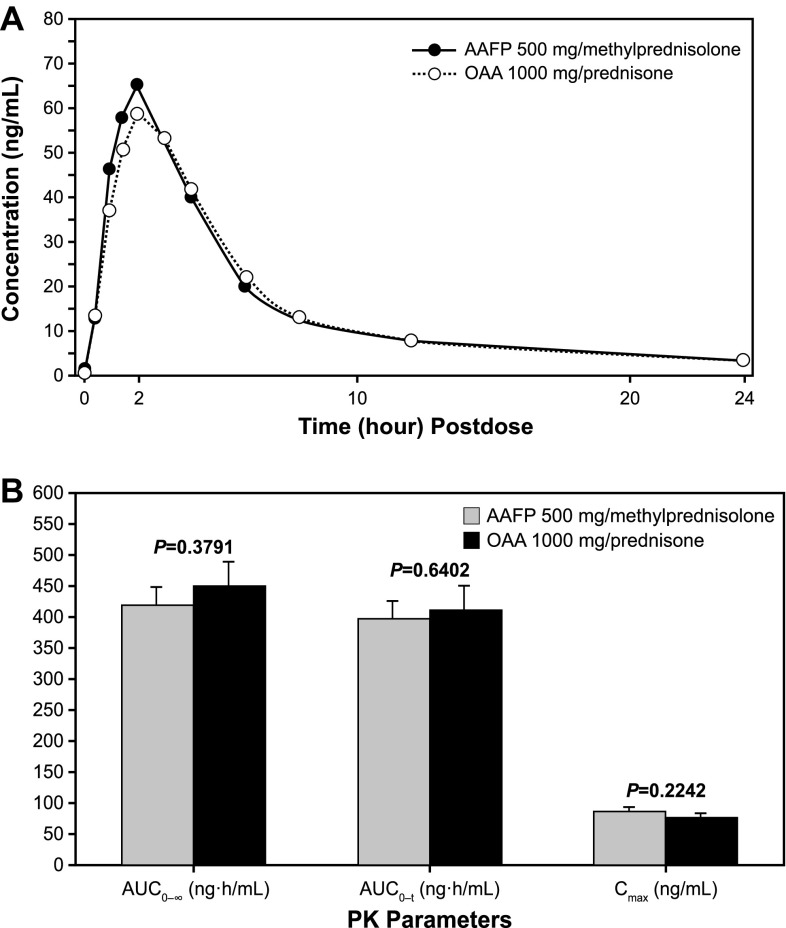
**a** Mean plasma abiraterone concentration–time plots on steady-state steroid and under fasted conditions for the pharmacokinetic population (linear scale). **b** AUC and *C*_max_ parameters (mean ± standard error) for AAFP 500 mg and OAA 1000 mg administered with steady-state methylprednisolone 4 mg and prednisone 5 mg, respectively. The *y* axis is intentionally unlabeled. *AAFP* abiraterone acetate fine particle, *AUC*_*0*–*∞*_ area under the plasma concentration–time curve from time 0 extrapolated to infinite time, *AUC*_0–*t*_ area under the plasma concentration–time curve from time 0 to the time of the last quantifiable concentration, *C*_max_ maximum concentration, *OAA* originator abiraterone acetate, *PK* pharmacokinetic

Following single-dose administrations of the test and reference drugs on a background of steady-state steroids, the average plasma concentration of abiraterone for AAFP closely overlapped the average plasma concentration for OAA, with the peak level of AAFP being slightly higher (Fig. 1a). Levels of both treatments peaked approximately 2 h postdose. This pattern was also observed with the individual subjects for each treatment (data not shown).

There were no statistically significant differences in the extent and rate of drug exposure between AAFP on a background of methylprednisolone versus OAA on a background of prednisone under fasted conditions (Table 2; Fig. 1b). There was a small but significant difference between AAFP 500 mg and OAA 1000 mg in mean *T*_½_ values for abiraterone (12.26 vs. 16.62 h, *P* < 0.001, respectively) (Table 2). With reference to OAA plus prednisone, the relative bioavailability of AAFP plus methylprednisolone as measured by percent of the test-to-reference mean was 93.3, 96.3, and 112.4% for AUC_0–∞_, AUC_0–*t*_, and *C*_max_, respectively.

**Table 2:** Pharmacokinetic parameters of AAFP 500 mg with methylprednisolone (4 mg BID) and OAA 1000 mg with prednisone (5 mg BID) under fasted conditions

Pharmacokinetic parameter	Statistic	AAFP 500 mg + methylprednisolone (4 mg BID) *N* = 36	OAA 1000 mg + prednisone (5 mg BID) *N* = 36
AUC_0–∞_ (ng·h/mL)	Mean	420.85	451.02
	SD	186.14	250.82
	Median	383.59	384.83
	Range (min–max)	109.11–845.97	140.84–1326.03
	CV (%)	44.23	55.61
AUC_0–*t*_ (ng·h/mL)	Mean	398.70	414.11
	SD	180.08	240.84
	Median	367.18	344.11
	Range (min–max)	99.43–773.55	122.59–1271.75
	CV (%)	45.17	58.16
*C*_max_ (ng/mL)	Mean	86.13	76.63
	SD	46.98	50.31
	Median	71.50	61.77
	Range (min–max)	32.24–205.63	18.50–236.86
	CV (%)	54.55	65.65
*T*_max_ (h)	Mean	1.99	2.17
	SD	1.14	0.92
	Median	2.00	2.00
	Range (min–max)	1.00–6.00	0.50–4.00
	CV (%)	57.25	42.37
*T*_½_ (h)	Mean	12.26	16.62
	SD	4.28	7.35
	Median	12.19	14.68
	Range (min–max)	4.54–26.63	5.80–43.46
	CV (%)	34.94	44.23

*AAFP* abiraterone acetate fine particle, *AUC*_*0*–*∞*_ area under the plasma concentration–time curve from time 0 extrapolated to infinite time, *AUC*_0–*t*_ area under the plasma concentration–time curve from time 0 to the time of the last quantifiable concentration, *BID* twice daily, *C*_max_ maximum concentration, *CV (%)* coefficient of variation expressed as percent, *OAA* originator abiraterone acetate, *SD* standard deviation, *T*_*½*_ terminal elimination half-life, *T*_max_ time of maximum concentration

### Bioequivalence analysis

**Fig. 2:**
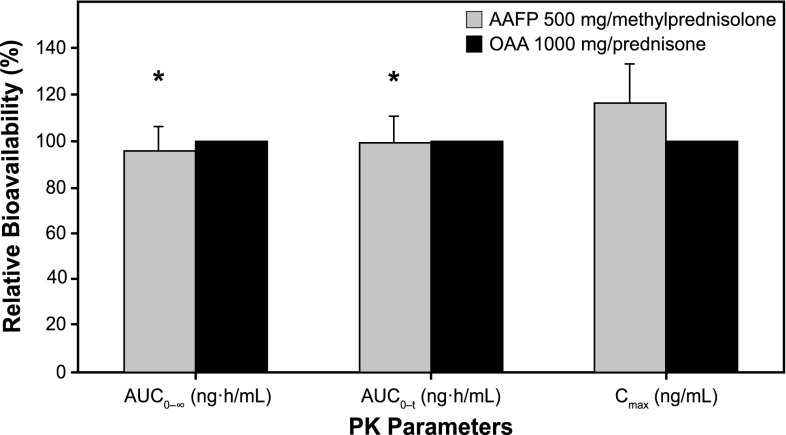
Relative bioavailability (%) (90% confidence interval) for AAFP 500 mg and OAA 1000 mg administered with steady-state methylprednisolone 4 mg and prednisone 5 mg, respectively. AAFP bars are normalized to 100% (reference OAA). *AAFP* abiraterone acetate fine particle, *OAA* originator abiraterone acetate, *PK*, pharmacokinetic. *Asterisk* 90% confidence interval met the requirement for bioequivalence (within 80.0–125.0%)

With reference to OAA 1000 mg, the relative bioavailability of AAFP 500 mg measured by the geometric mean ratio for AUC_0–∞_ was 95.9% (90% CI 86.0–106.9); for AUC_0–*t*_ was 99.2% (90% CI 88.7–110.9); and for *C*_max_ was 116.8% (90% CI 102.2–133.4) (Table 3; Fig. 2). These results indicate that a single dose of AAFP 500 mg on a background of steady-state methylprednisolone (4 mg BID) under fasted conditions was bioequivalent in terms of AUC to a single dose of OAA 1000 mg on a background of steady-state prednisone (5 mg BID) under fasted conditions based on the bioequivalence criteria of the 80–125% limits for the 90% CIs. For *C*_max_, the upper bound of the 90% CI fell slightly above the limit of predefined bioequivalence criteria for AAFP (Table 3).

**Table 3:** Bioequivalence analysis of relative bioavailability (pharmacokinetic population)

Parameter	Average (raw data)			ANOVA model-based least square mean (log scale)					
	AAFP 500 mg + methylprednisolone (4 mg BID)	OAA 1000 mg + prednisone (5 mg BID)	Ratio (%)	AAFP 500 mg + methylprednisolone (4 mg BID)	OAA 1000 mg + prednisone (5 mg BID)	Mean difference	90% CI	Geometric mean ratio	(90% CI of ratio)
AUC_0–∞_ (ng·h/mL)	420.851	451.020	103.3	5.939	5.981	−0.042	−0.151, 0.067	0.959	0.860, 1.069
AUC_0–*t*_ (ng·h/mL)	398.700	414.108	107.2	5.879	5.887	−0.008	−0.120, 0.104	0.992	0.887, 1.109
*C*_max_	86.127	76.634	130.2	4.315	4.160	0.155	0.022, 0.288	1.168	1.022, 1.334

*AAFP* abiraterone acetate fine particle, *ANOVA* analysis of variance, *AUC*_*0*–*∞*_ area under the plasma concentration–time curve from time 0 extrapolated to infinite time, *AUC*_0–*t*_ area under the plasma concentration–time curve from time 0 to the time of the last quantifiable concentration, *BID* twice daily, *C*_max_ maximum concentration, *CI* confidence interval, *OAA* originator abiraterone acetate

### Safety

Thirty-six of the 37 enrolled subjects received all planned doses of AAFP 500 mg and OAA 1000 mg per protocol. One subject who received AAFP in the first period of the study was lost to follow-up. Twenty mild (Grade 1) treatment-emergent AEs (TEAEs) were reported during the course of the study in 13 patients, of which 11 were considered by the investigator to be related to, and consistent with, the tested drugs. Table 4 shows the number and percentage of subjects with TEAEs occurring in ≥2 subjects in each system and organ class. No deaths or other serious TEAEs and no discontinuations due to a TEAE occurred during the study. No TEAEs related to clinical laboratory evaluations, vital signs, or electrocardiography were reported, and no clinically significant abnormal physical examination findings were reported. All TEAEs were considered to be resolved by the end of the study.

**Table 4:** Number and percentage of subjects with TEAEs by system and organ class (events occurring in ≥2 subjects in each category)

System and organ class	Any treatment *N* = 37
All TEAEs occurring in ≥2 subjects, *n* (%)	
Gastrointestinal disorders	2 (5.4)
General disorders and administration-site conditions	2 (5.4)
Infections and infestations	2 (5.4)
Injury, poisoning, and procedural complications	3 (8.1)
Nervous system disorders	3 (8.1)
Skin and subcutaneous tissue disorders	2 (5.4)

*TEAE* treatment-emergent adverse event

## Discussion

The present study demonstrated that AAFP on a steady-state background of methylprednisolone meets the bioequivalence criteria to OAA on a steady-state background of prednisone for AUC_0–∞_ and AUC_0–*t*_ variables. For *C*_max_, although the bioequivalence criteria were not met, the relative bioavailability was 112.4%, and this small difference was not statistically significant (*P* = 0.2242). The inherent properties of methylprednisolone, an alternative steroid to prednisone, did not negatively impact the mean values for AUC and *C*_max_. Thus, the administration of methylprednisolone with the AAFP formulation appeared to be well tolerated and safe, and it provided similar bioavailability compared with OAA and prednisone. Methylprednisolone has lower mineralocorticoid effects, while retaining similar glucocorticoid and anti-inflammatory effects to prednisone at the doses used in this study [13]. Based on the results of this study, this difference between methylprednisolone and prednisone does not appear to impact abiraterone exposure levels.

Importantly, compared with OAA and prednisone, the combination of AAFP and methylprednisolone resulted in a decrease in intersubject abiraterone drug level variability, as demonstrated by the lower CVs across all key parameters of AUC_0–∞_, AUC_0–*t*_, and *C*_max_. This may be due to the increased bioavailability of the new formulation, which halved the dose of abiraterone acetate required to achieve the same blood levels under fasted conditions [14].

Considerable variability has been reported for OAA, with between-subject variability in healthy subjects ranging from 40.5 to 140.6% for AUC_0–∞_ and from 32.7 to 119.8% for *C*_max_ [10]. Moreover, this variability has been demonstrated when OAA is administered to mCRPC patients in particular. An FDA clinical pharmacology review of OAA indicated significant intersubject variability after a single dose of OAA in mCRPC patients: CV 107% for AUC_24h_ and 140% for *C*_max_, and after multiple-day dosing, the CV was approximately 64% for AUC_24h_ and 79% for *C*_max_ [10]. Ryan et al. [8] reported that the CV for AUC_0–∞_ was 57.9% and for *C*_max_ was 71.9% in a sample (*n* = 6) of mCRPC patients administered the 1000-mg dose of OAA under fasted conditions.

It was previously observed that in conjunction with increasing the bioavailability of AA under fasted conditions, the AAFP formulation had a less dramatic food effect. This was demonstrated in a food effect study [15] that showed that the extent of AAFP 500-mg drug exposure, although still impacted by food, was considerably less than what was reported for OAA [10].

The improved variability finding is intriguing, especially considering the potential positive impact of the lower variability of abiraterone drug exposure on clinical outcomes, including suppression of testosterone. This is equally so with respect to safety and tolerability. For example, if the occurrence and frequency of hepatotoxicity is related to a high overall extent of exposure, a reduction in variability could decrease the number of patients with higher ranges of drug exposure, and thereby reduce their potential to develop this toxicity. High degrees of drug variability can challenge patients and clinicians, both in ensuring that efficacious drug levels are achieved in most or all patients, and in minimizing toxicity associated with excessively high blood drug levels [9].

The limitations of this study include the single dosing in healthy volunteers, and the fact that there was no randomization of the AA formulations to the alternative steroid (e.g., AAFP with prednisone and OAA with methylprednisolone). In addition, this is not a formal drug–drug interaction study, in which much higher doses of steroids might have demonstrated an impact on abiraterone concentration. The greatest strengths of this study are that the doses of prednisone and methylprednisolone were tested at levels that would typically be used clinically, and that the bounds of the CI of the geometric mean ratio of AUC of abiraterone for AAFP and OAA met the requirement for bioequivalence. An additional strength is the crossover design, allowing subjects to serve as their own control.

Further investigation of AAFP versus OAA is warranted in mCRPC patients over a longer duration of administration, including an evaluation of pharmacodynamic indicators such as testosterone levels, prostate-specific antigen levels, durability of response, and time to progression, in addition to abiraterone drug levels. Such a study (ClinicalTrials.gov Identifier: NCT02737332) is currently underway.

In conclusion, with regard to AUC, AAFP 500 mg administered on a background of steady-state methylprednisolone was shown to be bioequivalent to OAA 1000 mg on a background of steady-state prednisone. The findings support the clinical use of methylprednisolone with AAFP. Additionally, the between-subject variability in drug exposure parameters (AUC and *C*_max_) was consistently lower for AAFP 500 mg compared with OAA 1000 mg; this could potentially have a favorable impact on clinical outcomes. A comparative study is underway to better understand the pharmacodynamics and clinical characteristics of AAFP versus OAA.
